# Is psychosis a syndemic manifestation of historical and contemporary adversity? Findings from UK Biobank

**DOI:** 10.1192/bjp.2021.142

**Published:** 2021-12

**Authors:** Kamaldeep Bhui, Kristoffer Halvorsrud, Roisin Mooney, Georgina M. Hosang

**Affiliations:** Department of Psychiatry and Nuffield Department of Primary Care Health Sciences, University of Oxford; and Centre for Psychiatry, Queen Mary University of London, UK; National Institute of Health Research Applied Research Collaboration (NIHR ARC) North Thames, University College London; and Department of Psychiatry, University of Oxford, UK; Department of Psychiatry, University of Oxford, UK; Centre for Psychiatry, Wolfson Institute of Preventive Medicine, Queen Mary University of London, UK

**Keywords:** Psychosis, syndemic, inflammation, adversity, social deprivation

## Abstract

**Background:**

Psychosis is associated with many forms of adversity, deprivation and living in urban areas.

**Aims:**

To investigate whether psychosis is part of a syndemic of multiple adversities.

**Method:**

Drawing on UK Biobank (UKBB) data (Project ID: 57601), we sought to understand mechanisms by which childhood, recent/contemporary and place-based adversities might cluster and interact to be implicated in pathways by which psychoses evolve. We investigated the associations between adversities, potential mediating inflammatory markers and ICD-10 diagnoses (F20–F31) of psychotic disorders. We fitted logistic regression models initially including all relevant candidate variables and used backwards deletion to retain theoretically plausible and statistically significant (*P* < 0.05) associations with psychotic disorders. The candidate variables were entered in a partial least squares structural equation model (PLS-SEM) to test for syndemic interactions between risk factors. We tested whether the findings were sensitive to demographics, gender and ethnicity.

**Results:**

We fitted a PLS-SEM including psychosis as a syndemic outcome, and identified three latent constructs: lifetime adversity, current adversity and biomarkers. Factor loadings were above 0.30, and all structural paths were significant (*P* < 0.05). There were moderate associations between lifetime adversity and current adversity (standardised coefficient s.c. = 0.178) and between current adversity and biomarkers (s.c. = 0.227). All three latent constructs showed small but significant associations with psychosis (s.c. < 0.04). Lifetime adversity and current adversity were more strongly associated among ethnic minorities (combined) than White British people.

**Conclusions:**

Our findings stress the importance of interactions between childhood and contemporary adversities in preventive and therapeutic interventions for psychotic disorders, especially among ethnic minorities.

## Psychoses

Psychosis is a severe mental illness with a multifactorial aetiology. Psychosis has an incidence of around 1% in the population, and this is higher among Black, Asian and ethnic minorities, and in migrant populations and urban settings.^1–3^ People from lower socioeconomic and ethnic minority backgrounds typically experience a higher number of negative life events, more ‘generic life stressors’ of modern life (e.g. occupational, financial, relational) and greater psychological distress.^4–9^ Some evidence^8,10–12^ suggests that psychological distress stems from comparatively limited access to resources for coping, whether socioeconomic, intrapersonal, interpersonal or more place-based factors (e.g. ethnic density and related support) that might buffer the effects of deprivation.^13^ Disproportionate exposure to stressors, adversity and trauma may explain the higher incidence of psychosis in ethnic minorities.^14,15^ Little research investigates mechanisms by which adversity, discrimination and racism, for example, lead to poor health outcomes.^16,17^

Childhood adversity, such as abuse and parental death, has been consistently shown to double the odds of developing psychosis.^18^ Adversities can result in over-utilisation and depletion of limited resources (personal, family, and community assets) to tackle chronic stress and in the use of alternative and potentially unhelpful health behaviours (e.g. alcohol, drugs or cigarette smoking); the cumulative effects can trigger dysregulation of biological endocrine and inflammatory responses.^5,19^ Childhood trauma and adversity can lead to elevated inflammation, which is implicated in the aetiology of mental disorders.^20–22^ Inflammation is a potential mechanism by which psychoses may emerge.^23,24^ Thus, adverse childhood experiences may be driving higher risks of pronounced inflammation, related cognitive impacts and greater risks of psychosis and poor outcomes.^22,25,26^

This biosocial strain, or ‘allostatic load’, leads to *weathering*, premature ageing and chronic illness.^5^ A growing body of research suggests that adversity also leads to physical multimorbidity in psychosis.^27^ Yet, the precise nature and balance of influences from adverse childhood trauma, deprivation, unemployment, poor housing, negative health behaviours (alcohol intake, unhealthy diet) and inflammation remain uncertain. Emerging research on ‘syndemics’ offers a helpful theoretical framework to link these diverse influences, including the role of racism and ethnic discrimination.

## Syndemics

The term ‘syndemic’ was introduced by Singer in 1996^28^ to outline synergistically related epidemics that cluster in places and in people, with the interaction of adverse socioenvironmental contexts and risks. The concept of a ‘syndemic’ is different from conceptualisations of comorbidity or multimorbidity, in which diseases coincidentally occur together rather than sharing fundamental causes that reinforce each other. There are similarities with ecosocial and sociodevelopmental models of poor health and psychoses respectively.^29–31^ The first syndemic described by Singer in 1996^28,32^ showed linkages between substance misuse, violence and AIDS among people living in low-income urban environments. This first confirmed syndemic explained complex causation of multiple diseases through social and structural conditions such as poverty, marginalisation, gender inequality, malnutrition and stigma.^29^ Since then, the number of proposed syndemics has expanded^32^ to include mental health outcomes such as depression. For example, studies of Mexican immigrant women in the USA show linkages between violence, immigration, depression, type 2 diabetes and abuse.^33^ More recently, studies of ethnicity and psychosis propose that ethnic disparities may be better understood and tackled as a syndemic.^13^

Investigations of syndemics of psychosis are warranted, particularly in light of the recent realisation and intensified UK policy agenda^34^ of tackling the stark social, health, racial and spatial inequalities in the incidence, care and outcomes, including premature mortality, among people living with psychosis.^35,36^ We propose that a deeper understanding of the complex social, health, racial and spatial factors that drive health inequalities may be best understood through a ‘syndemic’ lens, followed by commensurate integrated care and policy.

This paper tests the complex interplay of risk factors such as psychosocial adversity (including adverse childhood experiences and discrimination, deprivation and demographics), inflammatory processes and psychoses. We also investigate whether the potential mechanisms in this interplay of influences might be relevant in explaining a higher risk of psychosis in ethnic minorities using a syndemic framework.

## Method

### The UKBB data-set

We used data from the UK Biobank (UKBB) (Project ID: 57601). This is an ongoing cohort collecting information about a range of background and health-related variables among more than 500 000 participants aged 40–69 years, recruited between 2006 and 2010.^37^ In accord with UKBB policies, any participants who subsequently withdrew were removed from our analyses.

We derived a dichotomous measure of psychosis meaning at least one ICD-10 diagnosis between F20 and F31; we derived subcategories of psychosis (i.e. non-affective: F20–29; affective: F30–31). These diagnoses were made for the majority on entry or at one of the follow-up interviews in UKBB (see https://biobank.ndph.ox.ac.uk/showcase/label.cgi?id=1712, and their timing). We used the variables from reporting sources consisting of ‘death register only’, ‘death register and other sources’, ‘primary care only’, ‘primary care and other source(s)’, ‘hospital admissions data only’, ‘hospital admissions data and other source(s)’, ‘self-report only’, ‘self-report and other source(s)’, as these options were mutually exclusive for each individual code. We assessed completion rates of relevant independent variables identified from previous syndemic models^38,39^ and in particular Beckie's heuristic model of allostatic load, health and health disparities.^5^

### Selected variables

#### Lifetime adversity (individual)

This was assessed using self-report items covering childhood adverse experiences (ACEs), intimate partner violence, sexual violence and war experience. The specific items were:

- felt hated by family member as a child
- felt loved by family member as a child (reverse coded)
- had someone to take them to a doctor when needed as a child (reverse coded)
- physically abused as a child
- sexually assaulted as a child
- ever victim of physically violent crime
- ever witnessed sudden violent death
- ever victim of sexual assault
- ever belittled by partner or ex-partner
- ever experienced physical violence by partner or ex-partner
- ever experienced sexual interference without consent
- ever been exposed to combat or war.

#### Current adversity (individual/household)

- Average total household income before tax (self-reported ordinal variable captured at baseline ranging from ‘less than £18 000’ to ‘greater than £100 000’: we reverse coded this for lower income categories to correspond to higher scores on the scale and therefore deprivation consistent with the direction of other variables)
- Member of leisure/social group (also reverse coded and self-reported at baseline)
- Age at recruitment (continuous based on date of birth)
- Felt very upset when reminded of stressful experience in past month (self-reported ordinal variable in online follow-up).

#### Current adversity (area-based)

Townsend deprivation index (ordinal variable ranging in UKBB from −6.25826 to 11.0013; higher scores mean more deprivation, relating the participant's postcode at baseline to: the proportion not owning a car, in overcrowded accommodation, not owner–occupier and unemployed).

#### Risky health behaviours

- Alcohol intake frequency (self-reported ordinal at baseline depending on frequency per day/week/month, reverse coded for higher frequency, corresponding to higher scores on scale and deprivation consistent with direction of other variables)
- Smoking status (self-reported ordinal at baseline with the categories ‘never’, ‘previous’ or ‘current’)
- Physical activity: International Physical Activity Questionnaire activity level (UKBB verified at baseline as ‘high’, ‘moderate’ or ‘low’ level of exercise, we reverse coded this)
- Poor appetite or overeating
- Trouble falling or staying asleep, or sleeping too much (self-reported measures dichotomised by UKBB in online follow-up)
- Lifetime number of sex partners (numeric self-reported at baseline)
- We dropped drug misuse as there were too few responses (<10 000) compared with other measures.

#### Biomarkers

- C-reactive protein (CRP) (measured by UKBB in mg/L)
- Albumin (g/L); cholesterol (mmol/L)
- Creatinine (umol/L); glucose (mmol/L)
- Glycated haemoglobin (HbA_1c_) (mmol/mol)
- Insulin-like growth factor 1 (IGF-1) (nmol/L)
- Triglycerides (mmol/L); pulse rate (bpm); diastolic and systolic blood pressure (automated readings in mmHg)
- White blood cell (leukocyte) count (10^9^ cells/L)
- red blood cell (erythrocyte) count (10^12^ cells/L)
- body mass index (BMI) (kg/m^2^)
- waist circumference (cm)
- hip circumference (cm)
- number of treatments and medications taken (self-reported)
- a diagnosis (1) or not (0) of diabetes (insulin dependent), hypertension (primary), and gastro-oesophageal reflux disease

All biomarker variables were clinically verified at baseline. These biomarkers were collected from the entire sample. Additional measures, including imaging and cognitive tests, were not considered in this paper as they were available only for a subsample.

### Statistical analyses

One approach to analysis is to include all variables in models irrespective of statistical significance, if plausibly implicated in studies of mechanisms.^13^ However, this is not the standard approach.^40^ In this paper we first included conceptually relevant variables implicated in the aetiology of psychosis. We then tested statistical associations with psychosis and used a significance criterion in a backwards stepwise deletion (*stepwise* command in Stata), entering all the above variables in a full model. We retained variables showing significance (we initially considered *P* < 0.050), and a more stringent and a more liberal cut-off of *P* < 0.010 and *P* < 0.100), respectively. The general approach to variable selection was to assemble as many conceptually and statistically relevant variables as possible, and so we wished to adopt the least stringent *P*-value while assuring reasonable model fit. After backwards elimination of candidate independent variables at *P* < 0.050 and *P* < 0.100, there was support only for variables with significant associations at *P* < 0.050 with a reported diagnosis of psychosis overall. Rather than select variables using overly restrictive statistical criteria of *P* < 0.010, we compared the findings with those when using *P* < 0.050 as the threshold for inclusion.

After non-significant variables were removed, their respective contributions to the regression models were predicted with seemingly unrelated estimation (SUEST),^41^ a method for testing comparability as unadjusted and adjusted odds ratios (ORs) with 95% confidence intervals (CIs). The SUEST test used in the models is appropriate in this context to estimate whether respective differences by ethnic and gender subgroups are significant. So with the ethnicity between-group comparisons as an example, our final model was first estimated separately on the White British and combined ethnic minority groups respectively, before the SUEST command in Stata was then invoked to enable cross-group comparisons estimating these two samples together and comparing whether any differences between estimates of the respective model variables were significant (through the deployment of a chi-squared test and its associated *P*-value).^42^ Several diagnostic tests of model fit were performed. This included the link test for model specification (interpreted as a correctly specified model if the parameter *_hat* in this test is significant (*P* < 0.050) while the parameter *_hatsq* is not significant).^43^

Then a goodness-of-fit (GOF) test was applied to logistic regression models that included one or more continuous independent variables. Incremental sample size principles were applied to a standardised Hosmer–Lemeshow test^44^ (with non-significant values set by *P* ≥ 0.050). This suggested reasonable model fit. Finally, tolerance values (the reciprocal of the variance inflation factor: 1/VIF) greater than 0.200 for included variables indicate no suspected multicollinearity issues.^43^

The retained variables from the logistic regressions were then featured in a structural equation model (SEM) using constructs (latent variables) that cannot be observed directly, but are each composed/explained by the different independent observable variables added to the model.^45^ These latent constructs enabled us to outline the various interactions in a syndemics framework (in path diagrams) including adversity, demographics, biomarkers and psychosis. Rather than a standard covariance-based SEM approach (CB-SEM) that would prerequisite the inclusion of continuous variables only, we opted for a bootstrapped partial least squares SEM (PLS-SEM) allowing the inclusion of both the continuous and discrete/categorical observed variables as the most suitable signifiers of our unobservable latent constructs.^40,46^

To check PLS-SEM quality, we considered factor loadings (>0.30 as minimal acceptance level^47^) of the observed variables associated with their latent constructs, as well as the significance (*P* < 0.050) of the standardised beta path coefficients between the overarching latent constructs. Following interpretation for the size of standardised coefficients, we interpreted a coefficient equal to or lower than 0.090 as a small effect, between 0.100 and 0.200 as a moderate effect and above 0.200 as a large effect.^43^ With the absolute GOF indices measuring the discrepancy between the proposed model and empirical covariance matrices of the data, researchers often concentrate on the relative fit owing to the inherent estimation inaccuracies in PLS-SEM (compared with CB-SEM).^48–52^ Support from available guidelines enabled a threshold to be set for interpreting relative GOF values equal to or higher than 0.900 as arguing in favour of the model.^51,53–56^

In addition, we conducted subgroup analyses by: gender (dichotomised by UKBB, with male as the reference) based on National Health Service records and self-report; and ethnicity (ethnic minorities aggregated; then Black, South Asian, White other, Other, and White British.

All analyses were conducted in Stata version 16.1 (Windows) by K.H. and reviewed with K.B.

Secondary analysis of UKBB data is covered by the study's ethical approval from the North West Multi-Centre Research Ethics Committee (ref.: 16/NW/0274; see: https://www.ukbiobank.ac.uk/learn-more-about-uk-biobank/about-us/ethics). All participants consented for data to be used in research and are able to withdraw consent at any point. Where this happened, we removed their data from the analyses and the reported findings.

## Results

Altogether, 480 participants were classified as having a psychotic disorder (i.e. at least one diagnosis between F20 and F31) and 133 976 participants were in the comparison group. The link (*_hat*: *P* = 0.012; *_hatsq*: *P* = 0.215), goodness-of-fit (*P* = 0.460) and multicollinearity (all 1/VIF values >0.430) tests all indicated promising results.

The final PLS-SEM included the following variables:

- felt hated in childhood
- ever victim of sexual assault
- household income
- poor appetite or overeating
- waist circumference
- hip circumference
- leukocyte count
- number of treatments and medications taken.

We present the background characteristics of the UKBB participants featuring in the final PLS-SEM analysis (in Tables 1 and 2).

**Table 1:** PLS-SEM Sample characteristics by gender

	Total sample (*n* = 134 456)	Gender	
		Male (*n* = 60 997)	Female (*n* = 73 459)
Age, years: mean (s.d.)	55.72 (7.74)	56.45 (7.80)	55.13 (7.64)
Ethnicity *n* (%)			
White British	122 135 (90.84)	55 804 (91.49)	66 331 (90.30)
White other	8298 (6.17)	3331 (5.46)	4967 (6.76)
Black groups	900 (0.67)	385 (0.63)	515 (0.70)
South Asian	840 (0.62)	494 (0.81)	346 (0.47)
Other ethnicity	1968 (1.46)	781 (1.28)	1187 (1.62)
Average household income <£18 000, *n* (%)	18 287 (13.60)	6968 (11.42)	11 319 (15.41)
Townsend (area) deprivation index, mean (s.d.)^a^	−1.69 (2.84)	−1.76 (2.84)	−1.64 (2.84)
Any psychosis reported (ICD-10 F20–F31), *n* (%)	480 (0.36)	226 (0.37)	254 (0.35)

PLS-SEM, partial least squares structural equation model.

a. Higher value denotes greater deprivation.

**Table 2:** PLS-SEM sample characteristics by ethnicity

	Ethnicity				
	White British (*n* = 122 135)	White other (*n* = 8298)	Black (*n* = 900)	South Asian (*n* = 840)	Other ethnicity (*n* = 1968)
Age, years: mean (s.d.)	55.93 (7.69)	54.46 (7.93)	50.29 (7.05)	52.10 (8.14)	52.10 (7.69)
Gender, *n* (%)					
Male	55 804 (45.69)	3331 (40.14)	385 (42.78)	494 (58.81)	781 (39.68)
Female	66 331 (54.31)	4967 (59.86)	515 (57.22)	346 (41.19)	1187 (60.32)
Average household income less than £18 000, *n* (%)	16 615 (13.60)	1015 (12.23)	154 (17.11)	113 (13.45)	332 (16.87)
Townsend (area) deprivation index, mean (s.d.)^a^	−1.83 (2.75)	−0.61 (3.22)	1.63 (3.47)	−0.55 (2.98	−0.02 (3.47)
Any psychosis reported (ICD-10 F20–F31), *n* (%)	403 (0.33)	50 (0.60)	7 (0.78)	3 (0.36)	14 (0.71)

PLS-SEM, partial least squares structural equation model.

a. Higher value denotes greater deprivation.

Table 3 and Table 4 outline the results of the logistic regression models as unadjusted ORs and adjusted ORs with associated confidence intervals. The tables show that the highest point estimate in the unadjusted analyses is represented by ‘ever victim of sexual assault’, with the odds of psychosis increasing by 99% for each score on the adversity measure (1.99–1.00). In the adjusted analyses, the highest estimate is ‘average total household income before tax’, with a 54% increased odds of reported psychosis for each lower bracket/category on the ordinal scale (1.54–1.00).

**Table 3:** Unadjusted logistic regressions with variables individually measured on reported diagnosis of psychosis

	Total sample, OR (95% CI)	Ethnicity, OR (95% CI)			Gender, OR (95% CI)		
		White British	Ethnic minority	*P*-value of SUEST test	Male	Female	*P*-value of SUEST test
Felt hated in childhood	1.57 (1.47–1.68)*	1.54 (1.43–1.66)*	1.60 (1.38–1.85)*	0.672	1.66 (1.49–1.84)*	1.55 (1.42–1.68)*	0.300
Ever exposed to combat or war	1.41 (0.98–2.01)	1.27 (0.83–1.95)	1.44 (0.71–2.92)	0.757	1.53 (1.02–2.28)*	0.96 (0.40–2.29)	0.333
Ever victim of sexual assault	1.99 (1.65–2.39)*	1.98 (1.62–2.43)*	1.79 (1.12–2.85)*	0.693	2.19 (1.57–3.05)*	2.04 (1.62–2.56)*	0.732
Age at recruitment	0.98 (0.98–0.99)*	0.98 (0.98–0.99)*	0.99 (0.98–1.00)*	0.844	0.98 (0.97–0.98)*	0.98 (0.98–0.99)*	0.173
Household income	1.96 (1.88–2.03)*	1.91 (1.83–1.99)*	2.13 (1.93–2.35)*	0.083	2.25 (2.13–2.38)*	1.73 (1.64–1.82)*	<0.001*
Poor appetite or overeating	1.84 (1.70–1.99)*	1.84 (1.69–2.00)*	1.77 (1.45–2.16)*	0.717	2.09 (1.86–2.35)*	1.72 (1.55–1.91)*	0.014*
Waist circumference	1.02 (1.02–1.03)*	1.02 (1.02–1.03)*	1.03 (1.02–1.03)*	0.084	1.02 (1.02–1.03)*	1.03 (1.03–1.03)*	0.027*
Hip circumference	1.02 (1.01–1.02)*	1.01 (1.01–1.02)*	1.02 (1.02–1.03)*	0.029*	1.01 (1.01–1.02)*	1.02 (1.02–1.02)*	0.092
Leukocyte count	1.04 (1.04–1.05)*	1.04 (1.03–1.05)*	1.12 (1.08–1.15)*	<0.001*	1.04 (1.03–1.05)*	1.05 (1.04–1.07)*	0.394
Number of treatments and medications	1.17 (1.16–1.18)*	1.17 (1.16–1.18)*	1.16 (1.14–1.18)*	0.268	1.17 (1.16–1.19)*	1.17 (1.16–1.18)*	0.583

SUEST, seemingly unrelated estimation.

* *P* < 0.050.

**Table 4:** Logistic regressions with variables adjusted for other model variables on reported diagnosis of psychosis

	Total sample, OR (95% CI)	Ethnicity, OR (95% CI)			Gender, OR (95% CI)		
		White British	Ethnic minority	*P*-value of SUEST test	Male	Female	*P*-value of SUEST test
Felt hated in childhood	1.28 (1.18–1.39)*	1.28 (1.17–1.40)*	1.20 (1.00–1.45)	0.570	1.27 (1.12–1.44)*	1.29 (1.16–1.43)*	0.865
Ever exposed to combat or war	1.13 (0.76–1.69)	1.11 (0.70–1.76)	1.11 (0.48–2.55)	0.995	1.16 (0.73–1.83)	0.95 (0.39–2.28)	0.692
Ever victim of sexual assault	1.34 (1.08–1.67)*	1.38 (1.09–1.75)*	1.09 (0.62–1.89)	0.432	1.34 (0.91–1.97)	1.41 (1.08–1.85)*	0.822
Age at recruitment	0.95 (0.94–0.96)*	0.95 (0.94–0.97)*	0.95 (0.92–0.98)*	0.770	0.94 (0.93–0.96)*	0.96 (0.95–0.98)*	0.101
Household income	1.54 (1.42–1.68)*	1.47 (1.34–1.62)*	1.94 (1.56–2.43)*	0.035*	1.77 (1.56–2.01)*	1.37 (1.22–1.53)*	0.007*
Poor appetite or overeating	1.34 (1.21–1.48)*	1.35 (1.21–1.51)*	1.32 (1.03–1.70)*	0.859	1.40 (1.20–1.64)*	1.31 (1.14–1.50)*	0.515
Waist circumference	1.03 (1.02–1.04)*	1.02 (1.01–1.03)*	1.03 (1.00–1.06)*	0.645	1.03 (1.01–1.05)*	1.02 (1.00–1.03)	0.352
Hip circumference	0.97 (0.95–0.98)*	0.97 (0.95–0.98)*	0.96 (0.93–1.00)*	0.842	0.96 (0.93–0.99)*	0.98 (0.96–1.00)	0.221
Leukocyte count	1.03 (1.01–1.06)*	1.03 (1.01–1.06)*	1.11 (1.01–1.22)*	0.114	1.03 (1.00–1.07)	1.04 (1.01–1.07)*	0.689
Number of treatments and medications	1.13 (1.10–1.16)*	1.13 (1.10–1.16)*	1.13 (1.05–1.22)*	0.990	1.12 (1.07–1.17)*	1.14 (1.10–1.18)*	0.421

SUEST, seemingly unrelated estimation.

* *P* < 0.050.

The retained variables for the logistic regression model were then investigated in the context of a PLS-SEM model. We combined ‘felt hated in childhood’, ‘victim of sexual assault’ and ‘experience of combat or war’ in a latent construct for lifetime adversity. Low household income, unhealthy diet and age contributed to current adversity. Age was included as ageing relates to a greater risk of poor health generally and although not strictly a form of adversity, the risks associated with ageing might best be captured as an immediate risk – reflecting age at the time of the study – rather than lifetime risk. Furthermore, in women, there is a second peak of risk at an older age.^57^ Waist circumference, hip circumference, high white blood cell count, and number of treatments and medications taken contributed to the latent construct of biomarkers. The initial results showed that the factor loadings for ‘experience of combat or war’ on the latent construct of lifetime adversity (0.235) and for age on current adversity (0.287) were both suboptimal, so were removed from the final PLS-SEM model.

The relative GOF statistic for the final model was high (0.900) and the standardised path coefficients between the latent constructs were all significant (*P* < 0.050), with a moderate direct effect size from lifetime and current adversity (0.178; the effect size in the reverse direction was 0.187, suggesting possible recall bias). There were large direct effects between current adversity and biomarkers (0.227; with 0.219 in the reverse direction, perhaps an indication of recall bias among those who are unwell). All other effects were small.

**Fig. 1:**
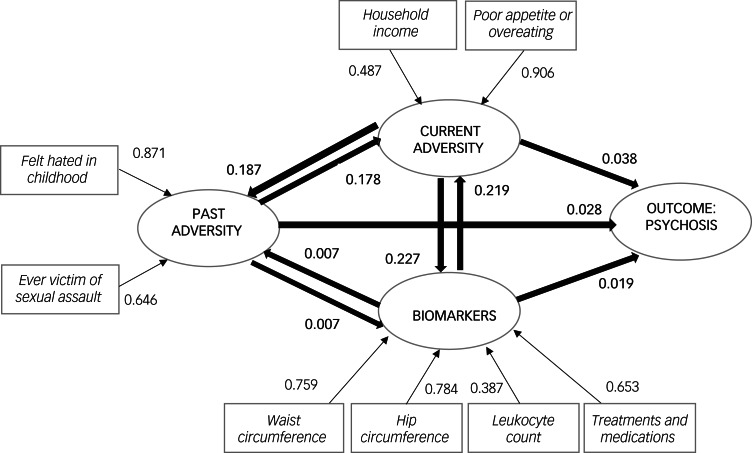
Proposed syndemics partial least squares structural equation model (PLS-SEM) for psychosis in the UK (showing direct effects). Latent constructs are shown in circles, observable variables in squares. The standardised coefficients between latent constructs (inner model) are depicted next to thicker arrows (directions of effects might go both ways), whereas factor loadings associated with latent constructs (outer model) are next to thinner arrows.

The main results are outlined in Table 5 (direct effects for total sample and subgroups) and Table 6 (combination of direct, indirect and total effects for total sample), and in Fig. 1.

**Table 5:** Syndemics PLS-SEM with direct effects in total sample and comparison by subgroups (ethnicity and gender)^a^

Structural comparison	Total sample, s.c. (*P*)	Ethnicity		Gender	
		White British (reference), s.c.	Ethnic minority (combined), s.c. (*P* compared with White British reference)	Male (reference), s.c.	Female, s.c. (*P* compared with male reference)
Past adversity → current adversity	**0.178 (*P* < 0.001)**	0.175	**0.203 (*P* = 0.027)**	0.154	0.155 (*P* = 0.144)
Past adversity → biomarkers	**0.007 (*P* = 0.048)**	0.009	0.006 (*P* = 0.806)	0.021	0.032 (*P* = 0.128)
Past adversity → psychosis	**0.028 (*P* < 0.001)**	0.026	0.028 (*P* = 0.855)	0.028	0.031 (*P* = 0.802)
Current adversity → past adversity	**0.187 (*P* < 0.001)**	0.184	**0.213 (*P* = 0.026)**	0.161	0.167 (*P* = 0.056)
Current adversity → biomarkers	**0.227 (*P* < 0.001)**	0.226	0.230 (*P* = 0.763)	0.213	**0.267 (*P* < 0.001)**
Current adversity → psychosis	**0.038 (*P* < 0.001)**	0.035	0.062 (*P* = 0.115)	0.052	**0.031 (*P* = 0.022)**
Biomarkers → past adversity	**0.007 (*P* = 0.048)**	0.009	0.006 (*P* = 0.801)	0.021	0.034 (*P* = 0.109)
Biomarkers → current adversity	**0.219 (*P* < 0.001)**	0.219	0.220 (*P* = 0.980)	0.208	**0.261 (*P* < 0.001)**
Biomarkers → psychosis	**0.019 (*P* = 0.048)**	0.017	0.034 (*P* = 0.180)	0.014	0.016 (*P* = 0.995)

PLS-SEM, partial least squares structural equation model; s.c., standardised coefficient; arrows indicate directions of associated tested.

a. **Bold** denotes significance at *P* < 0.050 (in total sample; or compared with White British (ethnicity); or male (gender)).

**Table 6:** Syndemics PLS-SEM for total sample showing combination of the direct, indirect and total effects

Structural comparison	Direct effects, s.c.	Indirect effects, s.c.	Total effects, s.c.
Past adversity → current adversity	0.178	0.016	0.194
Past adversity → biomarkers	0.007	0.041	0.047
Past adversity → psychosis	0.028	0.009	0.037
Current adversity → past adversity	0.187	0.017	0.205
Current adversity → biomarkers	0.227	0.020	0.247
Current adversity → psychosis	0.038	0.013	0.050
Biomarkers → past adversity	0.007	0.042	0.048
Biomarkers → current adversity	0.219	0.019	0.239
Biomarkers → psychosis	0.019	0.011	0.029

PLS-SEM, partial least squares structural equation model; s.c., standardised coefficient; arrows indicate directions of associated tested.

Only ‘poor appetite or overeating’ would have been dropped from the final PLS-SEM analysis had we used a more stringent cut-off of *P* < 0.010 instead of *P* < 0.050, and as a sensitivity check we ran the overall model without this variable to verify that similar results (significant associations) were retained for all other variables.

The subgroup analyses (also displayed in Tables 3–5) reveal some, but relatively few, significant modifications by ethnicity and gender. Perhaps most notable are the significantly greater effects of low household income in adjusted analyses and the paths between lifetime and current adversity for the combined ethnic minority category compared with the White British group.

## Discussion

### Summary of key findings

We have drawn on the available (and rapidly emerging) literature on syndemics and the cumulative effects of experiencing co-occurring adversities in the context of lifetime adversities (past and potentially ongoing). We examined the complex interplay between a range of potential explanatory variables as social and fundamental causes of psychosis and potentially of inequalities in experience of psychoses.^29^ There appear to be important links between lifetime adversity and current adversity, perhaps reflecting recall bias but likely also showing that the experience of adversity early in life will lead to later adversity also. Contemporary adversity was associated with raised inflammatory markers, more so than lifetime adversity, again suggesting an interplay between lifetime and present adversity and inflammation. The relationship between lifetime and present adversity was especially marked among ethnic minorities (aggregated group). Thus, findings suggest an overall model of psychosis that needs to consider early-life adversity as a preventive target as well as a therapeutic target for those who develop psychosis. Further work is needed on ethnicity and gender with larger samples. The small associations with psychosis are not surprising given that this is a low-incidence condition; the effects are not insignificant in the context of public health approaches to prevention, which shift the population distributions of risk factors to the left, to reduce the total samples that might develop a condition. In large populations, even small effect sizes may result in significant numbers of people developing psychosis and related disabilities.

A recent study^13^ found that ethnic inequalities in psychosis were better explained by a syndemic whereby harmful social and health conditions co-occur in geographical/temporal contexts. The study evidenced that the co-existence of crime and violence, mental health problems, substance misuse and risky sexual behaviours explained the risk of psychosis in a specific London borough (Hackney). It also showed that all ethnic groups were vulnerable to this area effect, for example White men in Hackney reporting comparatively more anxiety, depression and adverse health behaviours than White men in the general UK population. The authors argued that ethnic minority and other marginalised groups are more likely to live in lower income households, often concentrated in relatively deprived areas affected by these unfavourable socioeconomic conditions. We explored such findings at a national level through a combined measure representing childhood adversity, low household income, adverse health behaviour in addition to biomarkers as possible intermediate mechanisms associated with psychosis. However, we are limited by relying on national data and that a more extensive investigation and confirmation of the wider range of potential variables interacting with specific location parameters (such as in the Hackney study) was not possible. There was a low number of significant adverse health behaviour variables that our analyses could return. As a consequence, our (national) model had to be simplified and requires replication in specific locations and prospective developments.

### Implications for research

We propose that further analyses are necessary using larger data-sets, yet many cohorts do not include ACEs, inflammatory markers and ICD-10 diagnoses, which were a strength of the UKBB data. There are no standard approaches to testing syndemic models. We therefore had to consider alternative approaches and chose the methods that seemed transparent and suitable for large data-sets with many variables that may all be interacting. For example, a seminal paper on syndemics theory^32^ suggests that many researchers have used a ‘sum score’ to demonstrate the existence of syndemics. This is an approach whereby a syndemic is expressed via a combined variable containing the sum total of all candidate health risks experienced by the study participants. The problem with this approach is that all risks are seen as equal (only one variable exists in the regression model, for example). This both increases the chance of generating a statistically significant finding and masks the individual contributions of each candidate risk variable. Furthermore, these studies tend to focus predominantly on individual risk factors only, while neglecting the potential influence of multiple risk factors (as central to syndemics frameworks). For instance, variables such as level of area deprivation^32^ (which was also included in the present study) are not always considered, nor are life-course exposures.

The techniques applied in this study were logistic regression and SEM, but there are other statistical techniques previously employed to measure and describe syndemic effects.^32^ Our study has shown one possible way that synergies between components comprising measures of adversity and relatively high biomarker values may increase a person's risk of transition to a clinical diagnosis of psychosis. However, we realise that other factors not accounted for here might be at play, as for example environmental exposures such as stress have in previous studies been shown to interact with genetic risk.^58^ Additionally, owing to a lack of more optimal measures in the data-set, there are opportunities for future studies to assess the detrimental effects of experienced racism and discrimination, as well as the suspected role of and the extent to which social support mechanisms – known in some wider literature to exert a potentially preventive effect on adverse health outcomes^59^ – can help mediate or even significantly dampen syndemic effects.

The emerging research on syndemics suggests the importance of mixed methods to triangulate findings from multiple sources and unearth how large-scale social forces converse in a complex manner to shape the evolution of multiple conditions. Qualitative studies, and anthropological/ethnographic investigations in particular, can complement epidemiological data with richer, deeper and more place-based examinations of potential multiplicative and synergistic interactions, or the ‘microfoundations for macro-level observations’.^32^

### Limitations of this study

The total sample that could be included for our analyses was relatively modest compared with the overall UKBB sample (>500 000). Some questions relating to key variables could not been administered to all participants in the UKBB data-set because of online follow-ups requiring participants’ email addresses. For example, some were important measures in a hypothesised syndemic model (e.g. of traumatic experiences or adverse health behaviours). cross-examination of different variables for our analyses/models reduced numbers further by following conventions of a completer analysis (listwise deletion).^60,61^

The effects by specific ethnic groups and by gender were not marked, partly owing to limited power. Indeed, UKBB recruits healthy volunteers and shows some under-representation of ethnic minorities. As UKBB is a cross-sectional study, we cannot infer causality. Thus, replication is necessary in early intervention, prospective and ethnically enriched samples. Future studies should also test for place effects, which can be components of a syndemic model, although we did test for area deprivation.

Key variables had to be derived at baseline (between 2006 and 2010) either because they were only available at that time in the data-set, or the numbers that had responded to follow-up questions were markedly lower.

We acknowledge that reported diagnoses of psychosis were relatively low in the overall sample (and even lower by demographic subgroups). We also did not consider the timing of the diagnoses, given the small number of people with specific diagnoses; for example 68% of those receiving a schizophrnia diagnosis did so before enrolment, and 79% by 2010, the rest being diagnosed by 2017.

Furthermore, our markers of inflammation retained in the models might not naturally be expected to be inflammatory biomarkers but indicate a potential inflammatory role. For example, obesity, waist circumference and inflammation are associated in psychoses.^62^ Adversity variables (e.g. childhood adversity) were based on retrospective reports of past events and in some instances it is possible that there was ongoing adversity.^8,13^ The findings are best considered hypothesised associations rather than representing causal mechanisms, especially as it is well-known from the literature that mental illnesses are associated with raised inflammatory biomarkers.^29^

High leukocyte (white blood cell) count is a known marker for severity of inflammation and may signal an underlying problem such as trauma or stress and has also been associated with certain diseases, infections and allergies, with relatively high mortality rates in middle-aged populations.^63^ However, the fact that this biomarker, along with waist and hip circumference and number of treatments/medications, was significant in our analyses does not tell us why other known biomarkers seemed to have little predictive value. This speaks to a wider issue in the literature, where consensus is yet to be established on which biomarkers are necessary to investigate mechanisms in syndemics and allostatic load models.^5^ Although needing further work, the relative importance of biomarkers might depend on the specific health outcome under investigation.^5^

Syndemic theory argues for co-occurring disease entities and interacting risk factors in webs of causation. This is difficult to apply to psychosis for many reasons. Psychoses are diagnosed using symptom patterns and have fuzzy boundaries, and produce categories that are contested, especially across ethnic groups. Thus, a syndemic theory of psychotic disorders needs to accommodate the different diagnostic processes for distinct conditions, for example cancer or infectious disease, which more usually have physiological and anatomical alterations. Psychotic symptoms that do not reach a threshold for a diagnosis are associated with a higher risk of later diagnosis and more severe illness later, and might be part of a syndemic and a component of the mechanism leading to psychotic disorders. Strictly speaking, syndemics reflect co-occurring epidemics. We did not assess these nor separate illnesses. In this study, we have looked primarily at psychosis and inflammatory markers as proxies for comorbidity. Syndemics that have been described usually include behaviours such as violence or drug misuse as well as conditions like HIV. Thus, health behaviours may also be considered, as our study showed that disordered eating patterns were relevant and perhaps a manifestation of distress.

### Implications for policy and practice

Public health policies are clearly not meeting the needs of people and stark inequalities persist, perhaps as funding sources are focused on single outcomes and certain diseases receive predominant attention.^29^ Enhancing the effectiveness of public health and care interventions for syndemic frameworks may tackle systemic drivers and save costs for an already overstretched National Health Service currently fighting a global pandemic. Indeed, COVID-19 may also be a manifestation of similar complex causal pathways. Obviously, practitioners also need assistance in developing, testing and implementing integrated and systemic interventions in partnership with local government and social care agencies.^29^ The findings suggest that clinical interventions will need to recognise the social and structural drivers of psychosis and that past and ongoing adversity should be a target for both public health prevention efforts and therapies that recognise this complexity. Thus, syndemic policies and practices will need to evolve with the evidence, which will need to be nuanced regarding different types of psychosis, larger samples of ethnic groups, and comorbidities with other psychiatric and medical disorders.

## Data Availability

UKBB data are accessible to all researchers by registration and entering into an appropriate institutional agreement with UKBB.

## Supplementary material

For supplementary material accompanying this paper visit http://dx.doi.org/10.1192/bjp.2021.142. click here to view supplementary material
