# Supplementary material for: Is psychosis a syndemic manifestation of historical and contemporary adversity? Findings from UK Biobank
Source: Br J Psychiatry. 2021 Dec;219(6):686–94. doi: 10.1192/bjp.2021.142 (PMC8636607; doi:10.1192/bjp.2021.142)
Supplement: Supplementary file 1 [file S0007125021001422sup001.docx]

### UKBB Stata codes

Backwise elimination

stepwise, pr(.05): logistic psychosis income_household Townsend_deprivation_index felt_hated sexually_molested_as_child phys_abuse_by_fam_as_child victim_phys_violent_crime witness_sudden_viole_death victim_sexual_assault belittled_by_partner_ex phys_violen_by_partner_ex sexual_interfer_partner_ex upset_reminded_stress_mo alcohol_intake smoking_status sex_partners ipaq_activity poor_appeti_overeat sleep_problems crp albumin cholesterol creatinine glucose hba1c igf1 triglycerides bmi_bodysize_measures waist_circumference hip_circumference gastro_reflux_disease leukocyte pulse_rate dia_blood_pressure_auto_2 sys_blood_pressure_auto_2 hypertension_primary diabetes_insulin leisure_social_activity combat_war red_blood_cell_count felt_loved_as_child take_to_dr_as_child treatments_medications age_recruitment_06_10

Logistic regressions and SUEST (unadjusted)

logistic psychosis income_household if ethnic_minority_numeric==0

est store wb_income

logistic psychosis income_household if ethnic_minority_numeric==1

est store em_income

suest wb_income em_income

test [wb_income_psychosis]income_household=[em_income_psychosis]income_household

logistic psychosis treatments_medications if ethnic_minority_numeric==0

est store wb_treatments_medications

logistic psychosis treatments_medications if ethnic_minority_numeric==1

est store em_treatments_medications

suest wb_treatments_medications em_treatments_medications

test [wb_treatments_medications_psycho]treatments_medications=[em_treatments_medications_psycho]treatments_medications

logistic psychosis felt_hated if ethnic_minority_numeric==0

est store wb_felt_hated

logistic psychosis felt_hated if ethnic_minority_numeric==1

est store em_felt_hated

suest wb_felt_hated em_felt_hated

test [wb_felt_hated_psychosis]felt_hated=[em_felt_hated_psychosis]felt_hated

logistic psychosis hip_circumference if ethnic_minority_numeric==0

est store wb_hip_circumference

logistic psychosis hip_circumference if ethnic_minority_numeric==1

est store em_hip_circumference

suest wb_hip_circumference em_hip_circumference

test [wb_hip_circumference_psychosis]hip_circumference=[em_hip_circumference_psychosis]hip_circumference

logistic psychosis poor_appeti_overeat if ethnic_minority_numeric==0

est store wb_poor_appeti_overeat

logistic psychosis poor_appeti_overeat if ethnic_minority_numeric==1

est store em_poor_appeti_overeat

suest wb_poor_appeti_overeat em_poor_appeti_overeat

test [wb_poor_appeti_overeat_psychosis]poor_appeti_overeat=[em_poor_appeti_overeat_psychosis]poor_appeti_overeat

logistic psychosis waist_circumference if ethnic_minority_numeric==0

est store wb_waist_circumference

logistic psychosis waist_circumference if ethnic_minority_numeric==1

est store em_waist_circumference

suest wb_waist_circumference em_waist_circumference

test [wb_waist_circumference_psychosis]waist_circumference=[em_waist_circumference_psychosis]waist_circumference

logistic psychosis age_recruitment_06_10 if ethnic_minority_numeric==0

est store wb_age_recruitment_06_10

logistic psychosis age_recruitment_06_10 if ethnic_minority_numeric==1

est store em_age_recruitment_06_10

suest wb_age_recruitment_06_10 em_age_recruitment_06_10

test [wb_age_recruitment_06_10_psychos]age_recruitment_06_10=[em_age_recruitment_06_10_psychos]age_recruitment_06_10

logistic psychosis victim_sexual_assault if ethnic_minority_numeric==0

est store wb_victim_sexual_assault

logistic psychosis victim_sexual_assault if ethnic_minority_numeric==1

est store em_victim_sexual_assault

suest wb_victim_sexual_assault em_victim_sexual_assault

test [wb_victim_sexual_assault_psychos]victim_sexual_assault=[em_victim_sexual_assault_psychos]victim_sexual_assault

logistic psychosis leukocyte if ethnic_minority_numeric==0

est store wb_leukocyte

logistic psychosis leukocyte if ethnic_minority_numeric==1

est store em_leukocyte

suest wb_leukocyte em_leukocyte

test [wb_leukocyte_psychosis]leukocyte=[em_leukocyte_psychosis]leukocyte

logistic psychosis combat_war if ethnic_minority_numeric==0

est store wb_combat_war

logistic psychosis combat_war if ethnic_minority_numeric==1

est store em_combat_war

suest wb_combat_war em_combat_war

test [wb_combat_war_psychosis]combat_war=[em_combat_war_psychosis]combat_war

logistic psychosis income_household if ethnicity_agg_numeric==4

est store wo_income

suest wb_income wo_income

test [wb_income_psychosis]income_household=[wo_income_psychosis]income_household

logistic psychosis treatments_medications if ethnicity_agg_numeric==4

est store wo_treatments_medications

suest wb_treatments_medications wo_treatments_medications

test [wb_treatments_medications_psycho]treatments_medications=[wo_treatments_medications_psycho]treatments_medications

logistic psychosis felt_hated if ethnicity_agg_numeric==4

est store wo_felt_hated

suest wb_felt_hated wo_felt_hated

test [wb_felt_hated_psychosis]felt_hated=[wo_felt_hated_psychosis]felt_hated

logistic psychosis hip_circumference if ethnicity_agg_numeric==4

est store wo_hip_circumference

suest wb_hip_circumference wo_hip_circumference

test [wb_hip_circumference_psychosis]hip_circumference=[wo_hip_circumference_psychosis]hip_circumference

logistic psychosis poor_appeti_overeat if ethnicity_agg_numeric==4

est store wo_poor_appeti_overeat

suest wb_poor_appeti_overeat wo_poor_appeti_overeat

test [wb_poor_appeti_overeat_psychosis]poor_appeti_overeat=[wo_poor_appeti_overeat_psychosis]poor_appeti_overeat

logistic psychosis waist_circumference if ethnicity_agg_numeric==4

est store wo_waist_circumference

suest wb_waist_circumference wo_waist_circumference

test [wb_waist_circumference_psychosis]waist_circumference=[wo_waist_circumference_psychosis]waist_circumference

logistic psychosis age_recruitment_06_10 if ethnicity_agg_numeric==4

est store wo_age_recruitment_06_10

suest wb_age_recruitment_06_10 wo_age_recruitment_06_10

test [wb_age_recruitment_06_10_psychos]age_recruitment_06_10=[wo_age_recruitment_06_10_psychos]age_recruitment_06_10

logistic psychosis victim_sexual_assault if ethnicity_agg_numeric==4

est store wo_victim_sexual_assault

suest wb_victim_sexual_assault wo_victim_sexual_assault

test [wb_victim_sexual_assault_psychos]victim_sexual_assault=[wo_victim_sexual_assault_psychos]victim_sexual_assault

logistic psychosis leukocyte if ethnicity_agg_numeric==4

est store wo_leukocyte

suest wb_leukocyte wo_leukocyte

test [wb_leukocyte_psychosis]leukocyte=[wo_leukocyte_psychosis]leukocyte

logistic psychosis combat_war if ethnicity_agg_numeric==4

est store wo_combat_war

suest wb_combat_war wo_combat_war

test [wb_combat_war_psychosis]combat_war=[wo_combat_war_psychosis]combat_war

logistic psychosis income_household if ethnicity_agg_numeric==2

est store bl_income

suest wb_income bl_income

test [wb_income_psychosis]income_household=[bl_income_psychosis]income_household

logistic psychosis treatments_medications if ethnicity_agg_numeric==2

est store bl_treatments_medications

suest wb_treatments_medications bl_treatments_medications

test [wb_treatments_medications_psycho]treatments_medications=[bl_treatments_medications_psycho]treatments_medications

logistic psychosis felt_hated if ethnicity_agg_numeric==2

est store bl_felt_hated

suest wb_felt_hated bl_felt_hated

test [wb_felt_hated_psychosis]felt_hated=[bl_felt_hated_psychosis]felt_hated

logistic psychosis hip_circumference if ethnicity_agg_numeric==2

est store bl_hip_circumference

suest wb_hip_circumference bl_hip_circumference

test [wb_hip_circumference_psychosis]hip_circumference=[bl_hip_circumference_psychosis]hip_circumference

logistic psychosis poor_appeti_overeat if ethnicity_agg_numeric==2

est store bl_poor_appeti_overeat

suest wb_poor_appeti_overeat bl_poor_appeti_overeat

test [wb_poor_appeti_overeat_psychosis]poor_appeti_overeat=[bl_poor_appeti_overeat_psychosis]poor_appeti_overeat

logistic psychosis waist_circumference if ethnicity_agg_numeric==2

est store bl_waist_circumference

suest wb_waist_circumference bl_waist_circumference

test [wb_waist_circumference_psychosis]waist_circumference=[bl_waist_circumference_psychosis]waist_circumference

logistic psychosis age_recruitment_06_10 if ethnicity_agg_numeric==2

est store bl_age_recruitment_06_10

suest wb_age_recruitment_06_10 bl_age_recruitment_06_10

test [wb_age_recruitment_06_10_psychos]age_recruitment_06_10=[bl_age_recruitment_06_10_psychos]age_recruitment_06_10

logistic psychosis victim_sexual_assault if ethnicity_agg_numeric==2

est store bl_victim_sexual_assault

suest wb_victim_sexual_assault bl_victim_sexual_assault

test [wb_victim_sexual_assault_psychos]victim_sexual_assault=[bl_victim_sexual_assault_psychos]victim_sexual_assault

logistic psychosis leukocyte if ethnicity_agg_numeric==2

est store bl_leukocyte

suest wb_leukocyte bl_leukocyte

test [wb_leukocyte_psychosis]leukocyte=[bl_leukocyte_psychosis]leukocyte

logistic psychosis combat_war if ethnicity_agg_numeric==2

est store bl_combat_war

suest wb_combat_war bl_combat_war

test [wb_combat_war_psychosis]combat_war=[bl_combat_war_psychosis]combat_war

logistic psychosis income_household if ethnicity_agg_numeric==3

est store asian_income

suest wb_income asian_income

test [wb_income_psychosis]income_household=[asian_income_psychosis]income_household

logistic psychosis treatments_medications if ethnicity_agg_numeric==3

est store asian_treatments

suest wb_treatments_medications asian_treatments

test [wb_treatments_medications_psycho]treatments_medications=[asian_treatments_psychosis]treatments_medications

logistic psychosis felt_hated if ethnicity_agg_numeric==3

est store asian_felt_hated

suest wb_felt_hated asian_felt_hated

test [wb_felt_hated_psychosis]felt_hated=[asian_felt_hated_psychosis]felt_hated

logistic psychosis hip_circumference if ethnicity_agg_numeric==3

est store asian_hip_circumference

suest wb_hip_circumference asian_hip_circumference

test [wb_hip_circumference_psychosis]hip_circumference=[asian_hip_circumference_psychosi]hip_circumference

logistic psychosis poor_appeti_overeat if ethnicity_agg_numeric==3

est store asian_poor_appeti_overeat

suest wb_poor_appeti_overeat asian_poor_appeti_overeat

test [wb_poor_appeti_overeat_psychosis]poor_appeti_overeat=[asian_poor_appeti_overeat_psycho]poor_appeti_overeat

logistic psychosis waist_circumference if ethnicity_agg_numeric==3

est store asian_waist_circumference

suest wb_waist_circumference asian_waist_circumference

test [wb_waist_circumference_psychosis]waist_circumference=[asian_waist_circumference_psycho]waist_circumference

logistic psychosis age_recruitment_06_10 if ethnicity_agg_numeric==3

est store asian_age_recruitment_06_10

suest wb_age_recruitment_06_10 asian_age_recruitment_06_10

test [wb_age_recruitment_06_10_psychos]age_recruitment_06_10=[asian_age_recruitment_06_10_psyc]age_recruitment_06_10

logistic psychosis victim_sexual_assault if ethnicity_agg_numeric==3

est store asian_victim_sexual_assault

suest wb_victim_sexual_assault asian_victim_sexual_assault

test [wb_victim_sexual_assault_psychos]victim_sexual_assault=[asian_victim_sexual_assault_psyc]victim_sexual_assault

logistic psychosis leukocyte if ethnicity_agg_numeric==3

est store asian_leukocyte

suest wb_leukocyte asian_leukocyte

test [wb_leukocyte_psychosis]leukocyte=[asian_leukocyte_psychosis]leukocyte

logistic psychosis combat_war if ethnicity_agg_numeric==3

est store asian_combat_war

suest wb_combat_war asian_combat_war

test [wb_combat_war_psychosis]combat_war=[asian_combat_war_psychosis]combat_war

logistic psychosis income_household if ethnicity_agg_numeric==5

est store other_income

suest wb_income other_income

test [wb_income_psychosis]income_household=[other_income_psychosis]income_household

logistic psychosis treatments_medications if ethnicity_agg_numeric==5

est store other_treatments

suest wb_treatments_medications other_treatments

test [wb_treatments_medications_psycho]treatments_medications=[other_treatments_psychosis]treatments_medications

logistic psychosis felt_hated if ethnicity_agg_numeric==5

est store other_felt_hated

suest wb_felt_hated other_felt_hated

test [wb_felt_hated_psychosis]felt_hated=[other_felt_hated_psychosis]felt_hated

logistic psychosis hip_circumference if ethnicity_agg_numeric==5

est store other_hip_circumference

suest wb_hip_circumference other_hip_circumference

test [wb_hip_circumference_psychosis]hip_circumference=[other_hip_circumference_psychosi]hip_circumference

logistic psychosis poor_appeti_overeat if ethnicity_agg_numeric==5

est store other_poor_appeti_overeat

suest wb_poor_appeti_overeat other_poor_appeti_overeat

test [wb_poor_appeti_overeat_psychosis]poor_appeti_overeat=[other_poor_appeti_overeat_psycho]poor_appeti_overeat

logistic psychosis waist_circumference if ethnicity_agg_numeric==5

est store other_waist_circumference

suest wb_waist_circumference other_waist_circumference

test [wb_waist_circumference_psychosis]waist_circumference=[other_waist_circumference_psycho]waist_circumference

logistic psychosis age_recruitment_06_10 if ethnicity_agg_numeric==5

est store other_age_recruitment_06_10

suest wb_age_recruitment_06_10 other_age_recruitment_06_10

test [wb_age_recruitment_06_10_psychos]age_recruitment_06_10=[other_age_recruitment_06_10_psyc]age_recruitment_06_10

logistic psychosis victim_sexual_assault if ethnicity_agg_numeric==5

est store other_victim_sexual_assault

suest wb_victim_sexual_assault other_victim_sexual_assault

test [wb_victim_sexual_assault_psychos]victim_sexual_assault=[other_victim_sexual_assault_psyc]victim_sexual_assault

logistic psychosis leukocyte if ethnicity_agg_numeric==5

est store other_leukocyte

suest wb_leukocyte other_leukocyte

test [wb_leukocyte_psychosis]leukocyte=[other_leukocyte_psychosis]leukocyte

logistic psychosis combat_war if ethnicity_agg_numeric==5

est store other_combat_war

suest wb_combat_war other_combat_war

test [wb_combat_war_psychosis]combat_war=[other_combat_war_psychosis]combat_war

logistic psychosis income_household if sex_numeric==0

est store male_income

logistic psychosis income_household if sex_numeric==1

est store female_income

suest male_income female_income

test [male_income_psychosis]income_household=[female_income_psychosis]income_household

logistic psychosis treatments_medications if sex_numeric==0

est store male_treatments_medications

logistic psychosis treatments_medications if sex_numeric==1

est store female_treatments

suest male_treatments_medications female_treatments

test [male_treatments_medications_psyc]treatments_medications=[female_treatments_psychosis]treatments_medications

logistic psychosis felt_hated if sex_numeric==0

est store ma_felt_hated

logistic psychosis felt_hated if sex_numeric==1

est store fe_felt_hated

suest ma_felt_hated fe_felt_hated

test [ma_felt_hated_psychosis]felt_hated=[fe_felt_hated_psychosis]felt_hated

logistic psychosis hip_circumference if sex_numeric==0

est store ma_hip_circumference

logistic psychosis hip_circumference if sex_numeric==1

est store fe_hip_circumference

suest ma_hip_circumference fe_hip_circumference

test [ma_hip_circumference_psychosis]hip_circumference=[fe_hip_circumference_psychosis]hip_circumference

logistic psychosis poor_appeti_overeat if sex_numeric==0

est store ma_poor_appeti_overeat

logistic psychosis poor_appeti_overeat if sex_numeric==1

est store fe_poor_appeti_overeat

suest ma_poor_appeti_overeat fe_poor_appeti_overeat

test [ma_poor_appeti_overeat_psychosis]poor_appeti_overeat=[fe_poor_appeti_overeat_psychosis]poor_appeti_overeat

logistic psychosis waist_circumference if sex_numeric==0

est store ma_waist_circumference

logistic psychosis waist_circumference if sex_numeric==1

est store fe_waist_circumference

suest ma_waist_circumference fe_waist_circumference

test [ma_waist_circumference_psychosis]waist_circumference=[fe_waist_circumference_psychosis]waist_circumference

logistic psychosis age_recruitment_06_10 if sex_numeric==0

est store ma_age_recruitment_06_10

logistic psychosis age_recruitment_06_10 if sex_numeric==1

est store fe_age_recruitment_06_10

suest ma_age_recruitment_06_10 fe_age_recruitment_06_10

test [ma_age_recruitment_06_10_psychos]age_recruitment_06_10=[fe_age_recruitment_06_10_psychos]age_recruitment_06_10

logistic psychosis victim_sexual_assault if sex_numeric==0

est store ma_victim_sexual_assault

logistic psychosis victim_sexual_assault if sex_numeric==1

est store fe_victim_sexual_assault

suest ma_victim_sexual_assault fe_victim_sexual_assault

test [ma_victim_sexual_assault_psychos]victim_sexual_assault=[fe_victim_sexual_assault_psychos]victim_sexual_assault

logistic psychosis leukocyte if sex_numeric==0

est store ma_leukocyte

logistic psychosis leukocyte if sex_numeric==1

est store fe_leukocyte

suest ma_leukocyte fe_leukocyte

test [ma_leukocyte_psychosis]leukocyte=[fe_leukocyte_psychosis]leukocyte

logistic psychosis combat_war if sex_numeric==0

est store ma_combat_war

logistic psychosis combat_war if sex_numeric==1

est store fe_combat_war

suest ma_combat_war fe_combat_war

test [ma_combat_war_psychosis]combat_war=[fe_combat_war_psychosis]combat_war

Logistic regressions (adjusted)

logistic psychosis income_household treatments_medications felt_hated hip_circumference poor_appeti_overeat waist_circumference age_recruitment_06_10 victim_sexual_assault leukocyte combat_war

linktest, nolog

estat gof, group(130)

regress psychosis income_household treatments_medications felt_hated hip_circumference poor_appeti_overeat waist_circumference age_recruitment_06_10 victim_sexual_assault leukocyte combat_war

estat vif

by ethnic_minority, sort : logistic psychosis income_household treatments_medications felt_hated hip_circumference poor_appeti_overeat waist_circumference age_recruitment_06_10 victim_sexual_assault leukocyte combat_war

by ethnicity_agg, sort : logistic psychosis income_household treatments_medications felt_hated hip_circumference poor_appeti_overeat waist_circumference age_recruitment_06_10 victim_sexual_assault leukocyte combat_war

by gender, sort : logistic psychosis income_household treatments_medications felt_hated hip_circumference poor_appeti_overeat waist_circumference age_recruitment_06_10 victim_sexual_assault leukocyte combat_war

SUEST (adjusted)

logistic psychosis income_household treatments_medications felt_hated hip_circumference poor_appeti_overeat waist_circumference age_recruitment_06_10 victim_sexual_assault leukocyte combat_war if ethnic_minority_numeric==0

est store wb

logistic psychosis income_household treatments_medications felt_hated hip_circumference poor_appeti_overeat waist_circumference age_recruitment_06_10 victim_sexual_assault leukocyte combat_war if ethnic_minority_numeric==1

est store em

suest wb em

test [wb_psychosis]income_household=[em_psychosis]income_household

test [wb_psychosis]treatments_medications=[em_psychosis]treatments_medications

test [wb_psychosis]felt_hated=[em_psychosis]felt_hated

test [wb_psychosis]hip_circumference=[em_psychosis]hip_circumference

test [wb_psychosis]poor_appeti_overeat=[em_psychosis]poor_appeti_overeat

test [wb_psychosis]waist_circumference=[em_psychosis]waist_circumference

test [wb_psychosis]age_recruitment_06_10=[em_psychosis]age_recruitment_06_10

test [wb_psychosis]victim_sexual_assault=[em_psychosis]victim_sexual_assault

test [wb_psychosis]leukocyte=[em_psychosis]leukocyte

test [wb_psychosis]combat_war=[em_psychosis]combat_war

logistic psychosis income_household treatments_medications felt_hated hip_circumference poor_appeti_overeat waist_circumference age_recruitment_06_10 victim_sexual_assault leukocyte combat_war if ethnicity_agg_numeric==4

est store wo

suest wb wo

test [wb_psychosis]income_household=[wo_psychosis]income_household

test [wb_psychosis]treatments_medications=[wo_psychosis]treatments_medications

test [wb_psychosis]felt_hated=[wo_psychosis]felt_hated

test [wb_psychosis]hip_circumference=[wo_psychosis]hip_circumference

test [wb_psychosis]poor_appeti_overeat=[wo_psychosis]poor_appeti_overeat

test [wb_psychosis]waist_circumference=[wo_psychosis]waist_circumference

test [wb_psychosis]age_recruitment_06_10=[wo_psychosis]age_recruitment_06_10

test [wb_psychosis]victim_sexual_assault=[wo_psychosis]victim_sexual_assault

test [wb_psychosis]leukocyte=[wo_psychosis]leukocyte

test [wb_psychosis]combat_war=[wo_psychosis]combat_war

logistic psychosis income_household treatments_medications felt_hated hip_circumference poor_appeti_overeat waist_circumference age_recruitment_06_10 victim_sexual_assault leukocyte combat_war if ethnicity_agg_numeric==2

est store bl

suest wb bl

test [wb_psychosis]income_household=[bl_psychosis]income_household

test [wb_psychosis]treatments_medications=[bl_psychosis]treatments_medications

test [wb_psychosis]felt_hated=[bl_psychosis]felt_hated

test [wb_psychosis]hip_circumference=[bl_psychosis]hip_circumference

test [wb_psychosis]poor_appeti_overeat=[bl_psychosis]poor_appeti_overeat

test [wb_psychosis]waist_circumference=[bl_psychosis]waist_circumference

test [wb_psychosis]age_recruitment_06_10=[bl_psychosis]age_recruitment_06_10

test [wb_psychosis]victim_sexual_assault=[bl_psychosis]victim_sexual_assault

test [wb_psychosis]leukocyte=[bl_psychosis]leukocyte

test [wb_psychosis]combat_war=[bl_psychosis]combat_war

logistic psychosis income_household treatments_medications felt_hated hip_circumference poor_appeti_overeat waist_circumference age_recruitment_06_10 victim_sexual_assault leukocyte combat_war if ethnicity_agg_numeric==3

est store asian

suest wb asian

test [wb_psychosis]income_household=[asian_psychosis]income_household

test [wb_psychosis]treatments_medications=[asian_psychosis]treatments_medications

test [wb_psychosis]felt_hated=[asian_psychosis]felt_hated

test [wb_psychosis]hip_circumference=[asian_psychosis]hip_circumference

test [wb_psychosis]poor_appeti_overeat=[asian_psychosis]poor_appeti_overeat

test [wb_psychosis]waist_circumference=[asian_psychosis]waist_circumference

test [wb_psychosis]age_recruitment_06_10=[asian_psychosis]age_recruitment_06_10

test [wb_psychosis]victim_sexual_assault=[asian_psychosis]victim_sexual_assault

test [wb_psychosis]leukocyte=[asian_psychosis]leukocyte

test [wb_psychosis]combat_war=[asian_psychosis]combat_war

logistic psychosis income_household treatments_medications felt_hated hip_circumference poor_appeti_overeat waist_circumference age_recruitment_06_10 victim_sexual_assault leukocyte combat_war if ethnicity_agg_numeric==5

est store other

suest wb other

test [wb_psychosis]income_household=[other_psychosis]income_household

test [wb_psychosis]treatments_medications=[other_psychosis]treatments_medications

test [wb_psychosis]felt_hated=[other_psychosis]felt_hated

test [wb_psychosis]hip_circumference=[other_psychosis]hip_circumference

test [wb_psychosis]poor_appeti_overeat=[other_psychosis]poor_appeti_overeat

test [wb_psychosis]waist_circumference=[other_psychosis]waist_circumference

test [wb_psychosis]age_recruitment_06_10=[other_psychosis]age_recruitment_06_10

test [wb_psychosis]victim_sexual_assault=[other_psychosis]victim_sexual_assault

test [wb_psychosis]leukocyte=[other_psychosis]leukocyte

test [wb_psychosis]combat_war=[other_psychosis]combat_war

logistic psychosis income_household treatments_medications felt_hated hip_circumference poor_appeti_overeat waist_circumference age_recruitment_06_10 victim_sexual_assault leukocyte combat_war if sex_numeric==0

est store male

logistic psychosis income_household treatments_medications felt_hated hip_circumference poor_appeti_overeat waist_circumference age_recruitment_06_10 victim_sexual_assault leukocyte combat_war if sex_numeric==1

est store female

suest male female

test [male_psychosis]income_household=[female_psychosis]income_household

test [male_psychosis]treatments_medications=[female_psychosis]treatments_medications

test [male_psychosis]felt_hated=[female_psychosis]felt_hated

test [male_psychosis]hip_circumference=[female_psychosis]hip_circumference

test [male_psychosis]poor_appeti_overeat=[female_psychosis]poor_appeti_overeat

test [male_psychosis]waist_circumference=[female_psychosis]waist_circumference

test [male_psychosis]age_recruitment_06_10=[female_psychosis]age_recruitment_06_10

test [male_psychosis]victim_sexual_assault=[female_psychosis]victim_sexual_assault

test [male_psychosis]leukocyte=[female_psychosis]leukocyte

test [male_psychosis]combat_war=[female_psychosis]combat_war

PLS-SEM

plssem (Past > felt_hated victim_sexual_assault) /*

*/ (Current > income_household poor_appeti_overeat) /*

*/ (Biomarkers > treatments_medications waist_circumference hip_circumference leukocyte) /*

*/ (Psychoses > psychosis), /*

*/ structural(Current Past, /*

*/ Biomarkers Past, /*

*/ Psychoses Past, /*

*/ Biomarkers Current, /*

*/ Psychoses Current, /*

*/ Psychoses Biomarkers) /*

*/ boot(200) seed(123) stats correlate(lv)

estat total

plssem (Past > felt_hated victim_sexual_assault) /*

*/ (Current > income_household poor_appeti_overeat) /*

*/ (Biomarkers > treatments_medications waist_circumference hip_circumference leukocyte) /*

*/ (Psychoses > psychosis), /*

*/ structural(Current Past, /*

*/ Biomarkers Past, /*

*/ Psychoses Past, /*

*/ Biomarkers Current, /*

*/ Psychoses Current, /*

*/ Psychoses Biomarkers) /*

*/ group(ethnic_minority_numeric, reps(200) groupseed(123) method(bootstrap) alpha(.05) plot)

plssem (Past > felt_hated victim_sexual_assault) /*

*/ (Current > income_household poor_appeti_overeat) /*

*/ (Biomarkers > treatments_medications waist_circumference hip_circumference leukocyte) /*

*/ (Psychoses > psychosis), /*

*/ structural(Current Past, /*

*/ Biomarkers Past, /*

*/ Psychoses Past, /*

*/ Biomarkers Current, /*

*/ Psychoses Current, /*

*/ Psychoses Biomarkers) /*

*/ group(ethnicity_agg_numeric, reps(200) groupseed(123) method(bootstrap) alpha(.05) plot)

plssem (Past > felt_hated victim_sexual_assault) /*

*/ (Current > income_household poor_appeti_overeat) /*

*/ (Biomarkers > treatments_medications waist_circumference hip_circumference leukocyte) /*

*/ (Psychoses > psychosis), /*

*/ structural(Current Past, /*

*/ Biomarkers Past, /*

*/ Psychoses Past, /*

*/ Biomarkers Current, /*

*/ Psychoses Current, /*

*/ Psychoses Biomarkers) /*

*/ group(sex_numeric, reps(200) groupseed(123) method(bootstrap) alpha(.05) plot)
